# The Effects of Vestibular Rehabilitation and Manual Therapy on Patients with Unilateral Vestibular Dysfunction: A Randomized and Controlled Clinical Study

**DOI:** 10.3390/ijerph192215080

**Published:** 2022-11-16

**Authors:** Ana Sedeño-Vidal, Fidel Hita-Contreras, María Alharilla Montilla-Ibáñez

**Affiliations:** 1 Department of Health Sciences, Faculty of Health Sciences, University of Jaen, 23071 Jaen, Spain; 2 Otorhinolaryngology Service, University Hospital “City of Jaen”, 23007 Jaen, Spain

**Keywords:** dizziness, vestibular rehabilitation, physical therapy, postural balance, unilateral vestibular hypofunction

## Abstract

(1) Objective: To determine the effect of a directed vestibular rehabilitation therapy (VRT) program with manual therapy (MT) on dizziness-related disability and imbalance symptoms among patients with peripheral unilateral vestibular dysfunction. (2) Methods: Eighty patients (54.75 ± 1.34 years) were allocated either to a control group (*n* = 40), who underwent a directed VRT program, or to an experimental group (*n* = 40), who received the same program plus MT once a week/4 weeks. We assessed their level of disability (Dizziness Handicap Inventory, DHI), balance confidence (the Activities-specific Balance Confidence scale—16 items), postural balance (resistive multisensor platform), and the frequency and intensity of dizziness symptoms (visual analog scale). (3) Results: Post-intervention between-group improvements were observed regarding DHI total score and intensity in the experimental group (*p* < 0.001), as well as four weeks later. Six months after, the experimental group exhibited improvements in the center of pressure velocity with eyes open (*p* = 0.019), DHI total score (*p* = 0.001) and subscales (all *p* < 0.05), and intensity (*p* = 0.003) and frequency (*p* = 0.010) of dizziness. Balance confidence improvements were observed 1 month (*p* = 0.035) and 6 months (*p* = 0.038) post-intervention. (4) Conclusions: Directed VRT plus MT is a safe and beneficial intervention that speeds up recovery for patients suffering from dizziness and instability derived from unilateral vestibular dysfunction.

## 1. Introduction

Unilateral vestibular hypofunction is the partial or complete loss of function of one of the peripheral vestibular sensory organs and/or vestibular nerves. Unilateral vestibular hypofunction (UVH) can be caused by vestibular neuritis or Ménière’s disease but may also be caused by trauma, surgical intervention, ototoxic medication, age related vestibular hypofunction, or other lesions of the vestibulocochlear nerve or labyrinth [1]. The symptoms and signs of acute UVH are spinning or non-spinning vertigo with unsteadiness, nausea/vomiting and/or oscillopsia, and spontaneous peripheral vestibular nystagmus, which is direction-fixed and enhanced by the removal of visual fixation and reduced VOR function. The chronic symptoms are vertigo or dizziness, head motion intolerance, oscillopsia, disequilibrium, and gait/postural imbalance with a tendency to fall toward the presumably affected side and/or nausea. Injuries to the visual system and the neck can also affect the vestibular system and cause dizziness [1,2]. 

Vertigo and dizziness are two very frequent reasons for medical consultation, affecting 20–30% of the general population. They account for 5–10% of consultations in primary care and 4% of visits to the emergency services. Dizziness affects 30% of the population over 65 years of age [3]. According to a cross-sectional study carried out in Germany, the prevalence of peripheral vestibular hypofunction increases from 2.4% among middle-aged and younger adults to 32.1% in adults 79 years and older [4].

Vestibular rehabilitation therapy (VRT) is a common treatment for dizziness and balance problems, which has shown to be effective in adult patients, as it improves postural control, functional capacity, and quality of life [4]. Vestibular rehabilitation exercises, based on the protocols by Cooksey and Cawthorne, gaze stabilization exercises, coordination exercises, and postural control exercises reduce dizziness and improve postural stability and visual acuity during head movements in subjects with vestibular hypofunction [4,5]. Previous studies have assessed the effectiveness of VRT in vestibular disorders, concluding that there is moderate-to-strong evidence of VRT being an effective treatment for peripheral unilateral vestibular disorders, able to decrease symptoms of instability and improve vestibular function in the short- and medium-term [4,5,6,7,8]. While some patients continue to complain of dizziness even after the application of this treatment, several studies have demonstrated the short-term efficacy of manual therapy on dizziness associated with neck pain, headache, and other musculoskeletal disorders [9].

The vestibular and cervical proprioceptive systems receive afferent input from mechanoreceptors and the cervical neuromusculature, generating a relationship between structures, which, if altered, may induce a vertiginous syndrome [10]. The mechanoreceptors located in the joint capsule of the cervical facet joint, together with the neuromuscular receptors of the cervical musculature (connected with vestibular structures through the spinovestibular tract) provide proprioceptive information, which is essential to maintaining posture and stability [10,11]. This explains why subjects with cervical disorders experience dizziness or instability, as well as a decrease in sensory and motor control. A study by Hack et al. [11] found that the rectus capitis posterior minor muscle has a membranous anatomical connection with the dura mater. Its alteration may therefore affect the balance and postural control of the subject.

The main objective of the present study was to determine the effect of a directed VRT program with manual treatment on dizziness-related disability and imbalance symptoms in patients with unilateral vestibular dysfunction. The hypothesis was that a directed VRT-based intervention with manual treatment would have significant positive effects on dizziness-related disability in patients with unilateral vestibular dysfunction.

The secondary objective was to explore which changes occurred in the frequency and intensity of dizziness symptoms, fear of falling, subjective confidence on balance, and psychological distress between baseline and follow-ups after an intervention was implemented that involved a directed VRT program with manual treatment for patients with unilateral vestibular dysfunction 

## 2. Materials and Methods

This study was designed as a double-blind randomized controlled trial. It was registered with the Clinical Trials Registry (NCT04720872) and approved by the Committee for Medical Research Ethics of the Ciudad de Jaén Hospital (2018/657).

Participants were recruited between November 2020 and January 2022. Inclusion criteria were the following: subjects 18 years old and over who reported moderate or severe feelings of dizziness, measured by visual analog scale (VAS), during the last three weeks with a clinical diagnosis of peripheral unilateral vestibular hypofunction in the subacute or chronic phase performed by a specialist physician and confirmed by video Head Impulse Test (vHIT) with gain scores < 0.8. Exclusion criteria were the following: being afflicted by the diseases of the central nervous system, degenerative or tumorous disease, acute infection, morphological or functional alteration of the lower limbs and/or suffering morphological alterations of the cervical and/or suboccipital spine, neuromuscular disease or trauma that prevented them from performing the exercises and treatments proposed in the study, cognitive dysfunction (unable to follow instructions and/or fill in forms), positive extension–rotation test (Klein test), positive Rancurel test, or cerebrovascular alterations. We also excluded all subjects who were taking drugs that could affect auditory and vestibular functions. None of the patients enrolled in the study had previously undergone VRT treatment nor virtual reality-based procedures.

Physicians at the department of otolaryngology at Jaén University Hospital and Bulevar Health Center referred eligible patients to the interventionist otolaryngologist, who performed the clinical assessments of the patients and provided patients with information about the study and their participation in it. Written informed consent was obtained from all participants. The experimental intervention was carried out at the Bulevar Health Center facilities and complied with the Declaration of Helsinki.

The baseline clinical assessment of the subjects was based on the diagnostic criteria for Acute Unilateral Vestibulopathy in Evolution by the Bárány Society [12]: acute or subacute onset of sustained, spinning or non-spinning vertigo of moderate to severe intensity, with continuous symptoms for more than 3 h but not lasting for 24 h; spontaneous peripheral vestibular nystagmus that is direction-fixed and enhanced by the removal of visual fixation with a trajectory appropriate to the semicircular canal afferents involved; the unambiguous evidence of reduced vestibulo-ocular reflex function on the opposite direction of the fast phase of the spontaneous nystagmus; no evidence for acute central neurological symptoms or acute audiological symptoms, such as hearing loss, tinnitus or other otologic symptoms (otalgia, etc.); and no acute central neurological signs. Postural imbalance in the Romberg test, which increases with eyes closed, typically shows a tendency to fall toward the slow phase of nystagmus [12]. In order to quantitatively show the presence of vestibular system deficit, we included tests of the oculomotor system for an impaired vestibulo-ocular reflex gain by vHIT (smooth pursuit, saccadic eye movements), head-thrust test, and clinical test for dynamic visual acuity. The intervening otolaryngologist assessed all selected subjects prior to randomization. Participants were then randomly assigned (1:1 allocation). Allocation followed a computer-generated list of random numbers (www.random.org, accessed on 17 November 2020) and employed opaque and sealed envelopes. The otolaryngologist was blinded for group allocation during the processes of randomization and intervention. In turn, subjects were blinded for group allocation. Only the intervening physiotherapist was not blinded to group allocation and knew the distribution of the subjects but did not participate in the assessment process. A blinded outcome evaluator carried out the follow-up assessments. An uninvolved statistician entered the data into the database during the statistical analyses phase.

### 2.1. Intervention Protocol

For four weeks, both groups received the same protocol of VRT, overseen by a physiotherapist with accredited competency courses. The treatment group (TG) received one weekly manual therapy session. The manual therapy protocol was based on the application of the high velocity/low amplitude technique on the atlanto–occipital axis cervical segment [13] and a technique of inhibition and myofascial release of the suboccipital musculature, approximately 20 min long. This treatment is based on the neurophysiologic relationship between the muscle spindles of the upper cervical spine segments (C2–C3 segment). Cervical spine intrinsic muscles connect with afferent pathways to the vestibular nuclei via the spinovestibular tract [10,14]. The directed VRT consisted of a program with gaze stabilization exercises (adaptation and substitution exercises), habituation exercises, and exercises aimed to improve balance and gait and walking. Exercise sessions were 30 min long. Patients in the treatment group received the intervention once per week for four weeks. The control group (CG) did not receive any manual therapy, only the VRT directed intervention once weekly for four weeks (Table 1).

After four weeks of intervention, all patients received an exercise program and written instructions to continue performing the same vestibular exercises at home for six months. The patients performed the exercises twice daily. Each session comprised twenty repetitions per exercise. Patients were informed about the purpose of exercises, and compliance was monitored via a daily journal and phone interviews. Both groups received recommendations to engage in general physical activities, such as walking or cycling. A graphical representation of the study design is displayed in Supplementary Material Section S1.

The intervention was performed by the same physiotherapist, an individual experienced in vestibular and manual therapy, in order to eliminate inter-examiner variability. Exclusion criteria during the intervention were: developing, over the course of the treatment, acute crises of vertigo, headaches, and/or neck pain that contraindicated the application of the proposed treatments, as well as failure to attend at least 75% of the sessions and a lack of compliance with the at-home exercise program. Previous studies indicated that, for patients without important comorbidities and acute/subacute vestibular hypofunction, a weekly control for 2–3 weeks would be sufficient [15]. 

### 2.2. Outcomes (Measures)

There were four measurement time points: pre-intervention, immediately after the end of the intervention (4 weeks +/− 3 days), 1 month after the end of the intervention, and 6 months after the end of the intervention. Patients were contacted and interviewed via phone for a follow-up after the last examination. All examinations were carried out by an investigator who was blinded to treatment allocation.

The Dizziness Handicap Inventory (DHI) is a multidimensional self-assessment scale that assesses and quantifies the level of disability and handicap associated with dizziness. It was initially developed by Jacobson and Newman in 1990 [16] and has been culturally adapted to the Spanish language (Supplementary Material Section S2) [17]. The Spanish version showed high reliability (0.97) and internal consistency (Cronbach 0.89). It consists of 25 items divided into three separate subscales: physical (DHI-P), emotional (DHI-E), and functional (DHI-F). It is possible to use either the total score or the scores of the three subscales separately. Participants were required to complete all 25 questions using ‘yes’, ‘sometimes’, or ‘no’, which corresponded to 4, 2, or 0 points, respectively. Scores therefore range from 0 to 100, with a score of 100 indicating a high level of disability and handicap from symptoms of dizziness. For the functional and emotional aspects: values 0–14 indicate low level disability; moderate disability when 15–24; and severe when over 25 points. As for the physical aspect: values 0–9 indicate low level disability, moderate when 10–16, and severe when 17 points or more.

Balance confidence was assessed by the Activities-specific Balance Confidence scale-16 items (ABC-16). This questionnaire is frequently employed to assess the level of confidence when performing a specific task without losing balance, becoming unsteady, or falling down [18]. It consists of 16 questions with scores ranging from 0% (no confidence) to 100% (totally confident) and provides a total score, which is obtained by adding the ratings (0–160) and dividing by 16. A high percentage indicates a high level of self-confidence. The present study employed the Spanish version of the questionnaire (Supplementary Material Section S2), which has been previously validated [19].

To evaluate postural control and balance, a resistive multisensor platform and the Free-Step Standard 3.0 software were used (Sensor Medica, Rome, Italy). For posturography testing each patient was instructed to maintain an upright position on the platform during 30 s for each test, first with eyes closed and then open. The stabilometric variables were obtained under both eyes-open (EO) and eyes-closed (EC) conditions. Each test lasted 30 s, with a 1 min interval between tests. The parameters evaluated were as follows: the area covered by the center of pressure (CoP) displacements (S, mm^2^); the velocity of CoP movements (V, mm/s); the length of the stabilogram (L, mm); and the average displacements of the CoP in the mediolateral (X) and anteroposterior (Y) directions (mm).

The frequency of dizziness was recorded via a self-monitoring diary, which registered the days in which subjects experienced dizziness or imbalance each week, on a weekly seven-point scale ranging from 0 (not once in a week) to 7 (every day of the week). The intensity of dizziness was registered through the visual analog scale (VAS) with a range from 0 to 10. In the VAS, patients estimated the intensity of their symptoms related to dizziness, vertigo, and imbalance, with 0 indicating the lowest level of dizziness and 10 severe dizziness [20].

### 2.3. Statistical Analysis

Continuous variables were described using means and standard deviations. Intention-to-treat analyses were performed in order to compare the intervention group with the control group. Results are presented as mean differences with 95% confidence intervals. The SPSS software version 22.0 (SPSS Inc., Chicago, IL, USA) was used to perform all statistical analyses. All statistical tests were two-sided and assumed a 5% significance level.

Sample size was estimated using the G-power software (version 3.1.9.2; Heinrich Heine University, Dusseldorf, Germany), using as a reference the data reported by Fraix et al. [21], through a repeated measures ANOVA (between factors). Assuming a significance level of 5%, a power of 80%, an estimated 10% drop-out rate, two groups with four measurements, and an effect size of 0.398, in order to detect differences between the two groups of participants through the study, 30 patients per group were required for a total of 60 study subjects. 

A descriptive analysis of the clinical and sociodemographic baseline variables was carried out employing means and standard deviation, and between-group comparisons were established using the independent t-test for continuous data and the chi-squared test of independence for categorical data. Homogeneity among groups was also studied using Levene’s Test. Normality was examined by means of the Shapiro–Wilk test. A repeated measures analysis linear model was used for the intra-subject comparison of the outcome variables at each time-point. The analysis assessed the differences between variables based on treatment, time, and interaction. This analysis was assessed with a general linear model of repeated measures and the Bonferroni multiple comparison adjustment. Effect size was estimated using the eta-squared parameter (η^2^). Sphericity was verified using Mauchly’s test and Greenhouse–Geisser correction.

## 3. Results

**Figure 1:**
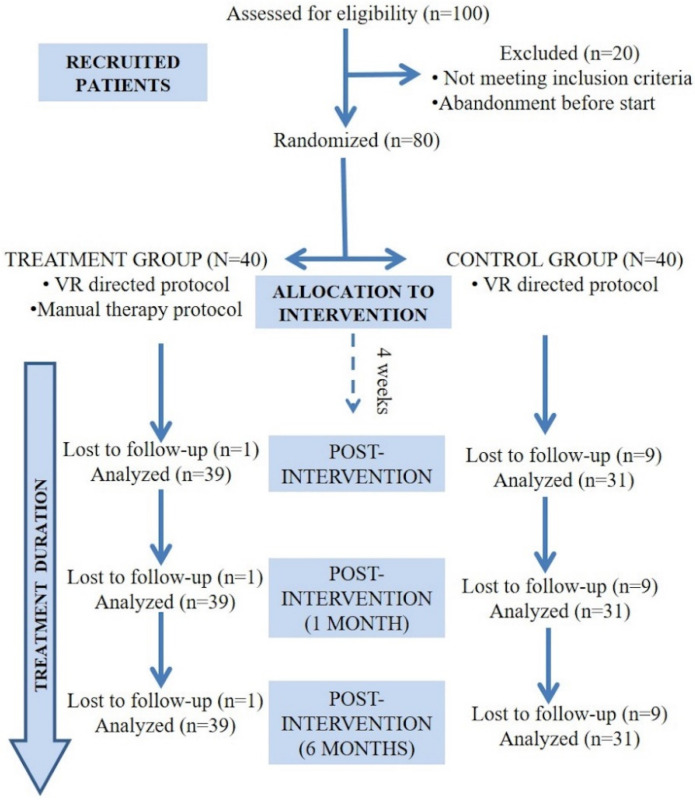
Flowchart diagram of participants.

A total of 80 patients were included in the study, 51 women (63.7%) and 29 men (36.3%) with a mean age of 54.75 ± 1.34 years and a 18–79 range. A total of 10 subjects (12.5%) were lost at follow-up (total subjects: *n* = 39 TG; *n* = 31 CG). There were no significant differences between groups regarding frequency or causes for dropping out. The causes of dropouts were the following: study duration, improvement in symptoms, or no change in symptoms (Figure 1). The characteristics of both groups are presented in Table 2. There were no significant baseline between-group differences regarding clinical and sociodemographic characteristics nor outcome measures. No adverse events of the intervention were registered. Adherence was >75% for all subjects included in the study. There was homogeneity in the variance between the groups and sphericity.

At baseline, the descriptive study of self-reported outcome measures indicated that the included participants had a moderate dizziness-related disability and severe symptoms of dizziness. The performance-based scores for balance and mobility indicated reduced balance when standing with eyes closed and reduced functioning in common mobility tasks (Table 2).

### 3.1. Dizziness-Related Disability

No significant between-group differences were found at baseline for the total score of the DHI questionnaire nor its subscales or domains (Table 2). For the main effect of the independent variable group, significant differences were observed for both the total DHI score (*p* = 0.001) and the three domains: DHI-E (*p* = 0.003), DHI-F (*p* = 0.001), and DHI-P (*p* = 0.021). Similarly, significant results were found for the main effect of the time-of-measurement variable for all subscales and the total DHI score (all *p* < 0.001). The analysis of between-group differences at the various times of measurement (Table 3) showed significant improvements in the experimental group for the DHI total score at the post-intervention (*p* < 0.001), 1 month post-intervention (*p* = 0.003), and 6 months post-intervention measurements (*p* = 0.004). The DHI also showed significant lower scores for the emotional, functional, and physical subscales of the TG compared with the CG at the post-intervention, 1 month post-intervention, and 6 months post-intervention measurements. Effect size was considered large for the total DHI score at 6 months (η^2^ = 0.11; Table 3).

### 3.2. Balance Confidence

No significant differences were found at baseline regarding the ABC-16 questionnaire (Table 2). Only one significant result was found for the main effect of the independent variable time (*p* < 0.001). Compared to the CG, the TG obtained significantly higher scores 1 month (*p* = 0.035) and 6 months (*p* = 0.038) after the end of the intervention period (Table 3). Effect size was considered medium at 6 months post-intervention (η^2^ = 0.068; Table 3).

### 3.3. Postural Control and Balance

No significant between-group differences were observed at baseline (pre-intervention) for any of the variables under the eyes-open and eyes-closed conditions (Table 3). The results only reveal significant changes according to group for the VEO variable (*p* = 0.004). The study of the main effects of the independent variables ‘time’ and ‘group’ yielded significant differences in all variables, both with eyes open and closed (*p* < 0.05).

The specific analysis of between-group differences (Table 3) showed no significant results post-intervention and only a significant improvement in the TG was observed for LEC (*p* = 0.029) at the 1 month post-intervention point. Meanwhile, at 6 months post-intervention, significant benefits were observed in the TG as compared with the CG regarding XEO (*p* = 0.009), YEO (*p* = 0.001), LEO (*p* = 0.006), VEO (*p* < 0.001), and SEO (*p* = 0.002), as well as for LEC (*p* = 0.031) and VEC (*p* = 0.019). The effect size was considered large and medium for all variables after 6 months (Table 3).

### 3.4. Frequency and Intensity of Dizziness

No significant between-group differences were detected at baseline concerning the frequency and intensity of dizziness (Table 3). The study of the main effects of the independent variables time and group yielded significant results for intensity (*p* < 0.001 and *p* = 0.002) and frequency (*p* < 0.001 and *p* = 0.019). The study of between-group differences (Table 3) showed statistically significant decreases regarding the frequency and intensity of dizziness in the TG, when compared with the CG at the post-intervention measurement (*p* = 0.038 and *p* < 0.001), after 1 month (*p* = 0.017 and *p* = 0.021), and after 6 months (*p* = 0.010 and *p* = 0.003). The effect size for frequency of dizziness was considered large at 6 months (η^2^ = 0.091 and η^2^ = 0.119; Table 3).

## 4. Discussion

The results yielded by this randomized controlled trial show that a four-week modified vestibular rehabilitation-based intervention with manual therapy improved dizziness-related disability and mobility, both post-intervention and in the follow-up. The effect of the intervention was maintained for six months after the end of the intervention. 

The results of our study show a positive effect of vestibular rehabilitation on postural stability, dizziness intensity, and the self-perception of disability and are in agreement with previous studies, which have demonstrated the benefits of VRT in peripheral unilateral vestibular hypofunction patients [1,21]. A recent Cochrane review [5] (2015) based on 39 randomized clinical trials and 2441 patients with unilateral vestibular dysfunction, concluded that VRT is a safe and effective treatment and showed the benefits of VRT over no treatment. This suggests that manual therapy could be a useful additional approach to reduce imbalance symptoms and to improve the quality of life of patients suffering from dizziness and vestibular disorders [9,22,23]. Moreover, the low drop-out rate incurred during the intervention period lends support to our hypothesis that a directed vestibular rehabilitation-based intervention with manual treatment has positive effects on dizziness-related disability in patients with unilateral vestibular dysfunction.

Dizziness is the most characteristic symptom of unilateral vestibular dysfunction and may be responsible for the low level of physical activity displayed by patients with this condition. This inactivity leads, in turn, to decreased physical function and disability [9]. A significant reduction in self-perceived disability after short- and medium-term interventions was observed in the present study. This finding could be related to the improvements in DHI scores. Our results are consistent with those of previous studies, which suggest that manual therapy is a beneficial approach to the treatment of patients with dizziness. Oriogo et al. [23] reported a VOR-gain remodulation immediately after the osteopathic manipulations, leading to a cessation of vertigo, which was maintained in the three months of follow-up. Pappa et al. [9] pointed out that, after a treatment session based on osteopathic manipulative treatment, the treatment group experienced an improvement in DHI total (*p* = 0.02), functional (*p* = 0.03), and physical (*p* = 0.03) scores when compared with a sham treatment group, as well as a reduction of swinging area (*p* = 0.02). A blinded, randomized, controlled comparative study conducted by Fraix et al. [24] in 2021 with participants assigned to four groups: osteopathic manipulative treatment alone; vestibular rehabilitation therapy alone; a combination of osteopathic manipulative treatment and vestibular rehabilitation therapy; and a nonintervention control group (with a total of three treatments lasting 45 min and 23 patients), reported that the combination group demonstrated significant improvement in DHI scores (*p* = 0.0284) between pre- and 3 month post-treatment measures and also observed clinically improved visual acuity in patients’ right eyes from pre- to post-treatment (*p* = 0.0325). These findings suggest that manual therapy could be a useful approach to reduce imbalance symptoms and to improve quality of life among patients suffering from dizziness.

The reduction in the frequency and intensity of dizziness and imbalance in patients with unilateral vestibular dysfunction after vestibular rehabilitation has been widely reported in the literature. Morozetti et al. [25] reported that 70% of patients treated with exercises based on a protocol of personalized vestibular rehabilitation had pre- and post-VRT values equal or higher than 50% at VAS, thus reaching ample statistical significance. These patients experienced significant improvements in their otoneurological clinical profile and their self-perception of dizziness.

Our study failed to find any significant mid-term increases in the Activities-specific Balance Confidence scale, which stands in contrast with previous studies involving other populations. A prospective cohort study carried out by Herdman et al. [8] in 116 patients diagnosed of unilateral vestibular hypofunction with at least two supervised VRT sessions showed improvement for every outcome measure. The percentage of patients who saw their ABC-16 scale outcomes improved or normalized reached 90.5%.

Morisod et al. [26] reported that, in 42 patients who underwent vestibular rehabilitation and post-rehabilitation posturography, the proportion of abnormal posturography significantly dropped from 79% to 33% (*p* < 0.001), and only pathological videonystagmography and a history of unilateral vestibular dysfunction remained significantly related to abnormal posturography. These results lend support to the findings of our study as far as postural variables are concerned.

The results of the present study suggest that directed VRT yields positive results in patients with unilateral vestibular dysfunction. Lilios et al. [27] showed that most randomized controlled trials (RCTs) report better outcomes after a supervised VRT treatment program regarding emotional status, dizziness, and balance. That particular systematic review failed to provide strong evidence that supervised programs surpass unsupervised protocols in patients with unilateral vestibular hypofunction because the self-reported subjective measures used by theRCTs included represent a serious limitation of their results. In that regard, previous studies support our results, with Shiozaki et al. [28] concluding that subjective dizziness decreased significantly regardless of whether supervised VRT was administered. However, dizziness evoked by head and body movements improved more significantly in the treatment group than in the control group. They suggested that supervised VRT could be highly effective in treating subjective dizziness in patients with chronic peripheral vestibular disorders, and that it benefited physical activity levels and the self-perception of subjective dizziness. Further studies with supervised and customized VRT and longer follow-up periods are needed in that regard.

Adherence is essential to clinical trials. In our study, 75 out of 80 subjects (93.75%) attended more than eight sessions. This high adherence may have contributed to the success of supervised treatment follow-up. Several factors may affect adherence treatment after vestibular dysfunction. Among such factors, not all individuals with unilateral vestibular hypofunction improved, regarding subjective and functional outcomes, when participating in VRT. Several studies have suggested that anxiety may contribute to either protracted or unsuccessful responses to VRT. MacDowell et al. [29] reported that participants with anxiety and/or depression took significantly longer to recover. Godeman et al. [30] found that anxiety correlated with persistent subjective symptoms one year after the onset of unilateral vestibular loss and that it was most frequent in female participants with dependent personalities and catastrophic thought. Anxiety traits and states, as well as somatization, appear to play a role in how patients perceive their handicap and symptoms. Other studies [8] have examined patient characteristics, such as age and time, from the onset of factors that might affect recovery, with conflicting results for both. Cousins et al. [31] reported that increased visual dependency, autonomic arousal, and anxiety predicted the poor outcomes of subjective complaints in patients with UVH but did not examine the effects of these factors on physical function. 

Some limitations justify caution when interpreting the results of this study. Only one therapist performed the manual therapy, and the results may not be generalizable to other therapists with a different training and experience profile. In order to increase the external validity of the findings and their generalizability we used strict eligibility criteria. The results of this study showed the positive effects of manual therapy in the management of patients with balance disorders. The general quality of the studies included was heterogeneous due to the scant literature available on the matter. Motivation and other psychological factors must be taken into account when interpreting results, especially concerning self-reported measures. Results must therefore be generalized with caution.

Nevertheless, the results from this study suggest that directed VRT plus manual therapy is a safe and beneficial intervention, which speeds up the recovery for patients with dizziness and instability derived from unilateral vestibular dysfunction. Our findings imply that directed VRT, implemented along with manual therapy, can be included in multidisciplinary rehabilitation programs as an effective treatment intervention compared with VRT alone. Further studies are required in order to secure outcomes that are more robust and examine how dose, the specifics of the progression of exercises, and the duration of the intervention period affect rehabilitation outcomes.

## 5. Conclusions

Directed vestibular rehabilitation plus manual therapy is a safe and beneficial intervention which speeds up recovery for patients with dizziness and instability derived from unilateral vestibular dysfunction and may be an effective treatment for patients with vertigo. Moreover, the results of this study support the hypothesis that directed vestibular rehabilitation and manual therapy can improve balance in individuals with dizziness. 

## Figures and Tables

**Table 1:** Exercise program.

EXERCISE
A card with a word was placed in front of patients. They moved their head for 1 min from side to side, maintaining the word in their field of vision. The same routine was performed for another minute while patients moved their head up and down. While the investigator held a card with a visible word, patients moved their head and hand in opposite horizontal directions maintaining the word in their field of vision for 1 min. The same routine was performed for another minute while patients moved their head and hand up and down. Patients looked at the investigator’s finger while sitting on a chair and turning their body from side to side. Patients were asked to move their eyes first slowly and then quickly in horizontal as well as vertical directions. These eye movements were performed in a sitting position. Patients tossed a ball from hand to hand and then up and down while following the ball with their eyes in a standing position. Patients rotated their heads along the horizontal and vertical plane and tilted their heads while fixing their eyes on a point, with eyes both open and closed. Patients rotated their heads from left to right, up and down, and from side to side. This was performed first slowly and then fast. Patients were seated on a chair while fixing their eyes on a point in front of them. They were instructed to move their trunk while maintaining a constant head position.
Patients had to move their trunk anteriorly to place a small object on the floor with rightward and leftward flexion and pass a ball from hand to hand behind their knee. Patients had to move from the sitting to the standing position and then turn around. Patients circled around a chair and climbed up and down a platform with eyes both open and closed.
Patients stood on one leg with their eyes first open then closed. This was performed while standing on a foam exercise mat. Patients stood on a firm surface with feet shoulder-width apart while looking straight ahead at a point on the wall. Their base of support was then reduced: from feet apart to feet together and then one foot in front of the other. They were required to walk and perform mental exercises at the same time.

**Table 2:** Score on all outcome measures per treatment group.

Outcomes	Treatment Group				Control Group			
	Baseline N = 40	Post-Treatment N = 39	1 Month N = 39	6 Months N = 39	Baseline N = 40	Post-Treatment N = 31	1 Month N = 31	6 Months N = 31
DHI total score DHI-Emotional DHI-Functional DHI-Physical	56.60 ± 22.57 15.60 ± 9.89 17.25 ± 9.80 15.75 ± 5.99	35.30 ± 22.83 10.05 ± 9.54 13.00 ± 9.40 12.25 ± 6.34	31.35 ± 24.31 9.40 ± 9.56 11.70 ± 9.69 10.25 ± 7.01	26.37 ± 24.41 8.80 ± 0.41 9.17 ± 9.33 8.40 ± 6.90	59.93 ± 19.08 19.16 ± 7.60 22.48 ± 8.32 18.32 ± 5.72	55.09 ± 20.41 17.48 ± 7.46 21.41 ± 8.02 16.19 ± 7.14	47.54 ± 17.94 15.80 ± 7.41 18.19 ± 7.04 13.54 ± 6.72	42.12 ± 17.97 13.48 ± 6.9 16.51 ± 7.26 12.12 ± 6.88
ABC-16	66.01 ± 21.19	72.10 ± 19.67	75.66 ± 20.59	78.72 ± 18.62	60.50 ± 16.828	64.11 ± 16.13	66.19 ± 14.11	69.93 ± 12.66
ID	6.52 ± 2.02	4.65 ± 2.04	4.10 ± 2.01	3.15 ± 2.23	6.90 ± 1.37	6.25 ± 1.46	5.16 ± 1.67	4.64 ± 1.78
FD	6.07 ± 1.81	4.70 ± 2.47	3.72 ± 2.27	2.65 ± 2.53	6.48 ± 1.33	5.80 ± 1.75	5.00 ± 2.04	4.19 ± 2.33
XEO (mm)	19.85 ± 18.36	18.27 ± 24.81	16.67 ± 25.88	9.05 ± 6.40	21.43 ± 15.35	22.64 ± 19.06	15.11 ± 12.02	19.15 ± 17.50
YEO (mm)	27.45 ± 37.39	13.90 ± 20.64	14.98 ± 23.47	8.55 ± 5.15	17.41 ± 19.32	21.42 ± 30.63	12.25 ± 12.69	15.54 ± 14.46
LEO (mm)	389.13 ± 243.17	356.79 ± 335.79	314.66 ± 253.31	227.70 ± 56.64	355.89 ± 118.19	374.81 ± 207.19	304.69 ± 92.41	304.00 ± 95.12
VEO (mm/s)	17.30 ± 11.26	14.32 ± 10.78	13.43 ± 7.90	11.09 ± 2.38	17.82 ± 11.97	14.71 ± 7.43	13.04 ± 5.37	13.50 ± 3.89
SEO (mm^2^)	221.91 ± 324.39	226.40 ± 471.17	247.41 ± 556.23	79.57 ± 96.75	153.62 ± 221.55	403.30 ± 618.45	201.90 ± 283.30	159.10 ± 165.16
XEC (mm)	36.86 ± 34.58	31.23 ± 29.71	22.95 ± 25.02	18.30 ± 17.34	28.79 ± 24.31	23.56 ± 16.87	20.73 ± 16.32	21.40 ± 18.41
YEC (mm)	43.55 ± 44.87	24.29 ± 25.54	22.19 ± 33.62	14.03 ± 15.06	20.86 ± 24.40	22.57 ± 37.39	21.15 ± 24.91	20.93 ± 19.59
LEC (mm)	496.47 ± 420.11	405.35 ± 256.51	303.70 ± 98.87	281.38 ± 115.80	422.57 ± 172.62	439.10 ± 316.28	355.35 ± 93.21	368.25 ± 210.22
VEC (mm/s)	23.39 ± 17.62	18.76 ± 14.90	14.91 ± 9.38	12.51 ± 3.41	16.32 ± 6.03	16.66 ± 10.67	14.52 ± 5.60	16.42 ± 9.46
SEC (mm^2^)	566.85 ± 727.07	460.18 ± 747.26	249.35 ± 370.88	173.33 ± 265.52	623.27 ± 858.47	570.58 ± 1027.85	250.70 ± 218.57	256.93 ± 217.84
Demographic measures at baseline								
Women (%) * Age (years) **	24 (60.00) 57.00 ± 13.04				27 (67.50) 56.50 ± 12.34			

Values expressed as mean ± standard deviation and frequency (percentage). ABC-16: Activities Specific Balance Confidence Scale—16 items; DHI: Dizziness Handicap Inventory total score; EO: eyes open; EC: eyes closed; FD: frequency of dizziness; ID: intensity of dizziness; L: length of the stabilogram described by the movements of the center of pressure; S: surface described by the movements of the center of pressure; V: velocity of displacement of the center of pressures; X: deviation of the center of pressure in the mediolateral directions; Y: deviation of the center of pressure in the anteroposterior axis. * No differences at baseline between sexes (*p* = 0.200). ** No differences at baseline between ages (*p* = 0.590).

**Table 3:** Main differences for each outcome between the study groups.

Outcome	Mean Difference (95% CI)	*p*-Value	Effect Size	Mean Difference (95% CI)	*p*-Value	Effect Size	Mean Difference (95% CI)	*p*-Value	Effect Size	Mean Difference (95% CI)	*p*-Value	Effect Size
	Baseline			Post-Intervention			1 Month after Intervention			6 Months after Intervention		
DHI total score	13.33 (3.24–23.42)	0.060	0.092	19.79 (9.38–30.21)	0.000	0.172	16.19 (5.80–26.59)	0.003	0.123	15.75 (5.32–26.18)	0.004	0.116
DHI- Emotional	5.56 (1.36–9.75)	0.100	0.010	7.43 (3.27–11.58)	0.001	0.001	6.40 (2.25–10.55)	0.003	0.003	4.68 (0.66–8.70)	0.023	0.023
DHI- Functional	5.20 (0.81–9.58)	0.210	0.075	8.41 (4.20–12.63)	0.000	0.000	6.49 (2.36–10.62)	0.002	0.002	7.34 (3.28–11.39)	0.001	0.001
DHI- Physical	2.57 (−0.23–5.38)	0.072	0.046	3.94 (0.74–7.14)	0.016	0.081	3.298 (0.00–6.58)	0.049	0.055	3.72 (0.43–7.02)	0.023	0.069
ABC-16	−5.50 (−14.77–−3.76)	0.240	0.020	−7.99 (−16.69–0.70)	0.071	0.046	−9.47 (−18.09–−0.84)	0.032	0.065	−8.79 (−16.59–−0.99)	0.028	0.068
ID	0.37 (0.46–−1.22)	0.376	0.011	1.60 (0.74–2.47)	0.000	0.166	1.06 (0.16–1.95)	0.021	0.075	1.49 (0.51–2.47)	0.003	0.119
FD	0.40 (−0.36–1.18)	0.297	0.016	1.10 (0.06–2.15)	0.038	0.061	1.27 (0.23–2.31)	0.017	0.080	1.54 (0.37–2.71)	0.010	0.091
XEO (mm)	1.58 (−6.62–9.79)	0.701	0.002	4.36 (−6.42–15.14)	0.422	0.009	−1.56 (−11.60–8.47)	0.757	0.001	10.09 (4.06–16.13)	0.001	0.141
YEO (mm)	−10.04 (−24.80–4.73)	0.179	0.026	7.52 (−4.74–19.78)	0.225	0.022	−2.73 (−12.08–6.61)	0.561	0.005	6.99 (2.02–11.97)	0.006	0.104
LEO (mm)	−33.22 (−128.31–61.83)	0.488	0.007	18.02 (−119.43–155.47)	0.794	0.001	−9.97 (−105.55–85.61)	0.836	0.001	76.29 (39.77–112.82)	0.000	0.204
VEO (mm/s)	53.52 (17.89–89.14)	0.400	0.117	0.38 (−4.15–4.92)	0.865	0.000	−0.38 (−3.70–2.93)	0.818	0.001	2.41 (0.90–3.92)	0.002	0.131
SEO (mm^2^)	−68.29 (−204.49–67.90)	0.321	0.015	176.89 (−82.91–436.71)	0.179	0.026	−45.51 (−264.65–173.63)	0.680	0.003	79.52 (16.43–142.61)	0.014	0.085
XEC (mm)	−8.06 (−22.70–6.56)	0.275	0.017	−7.66 (−19.6–4.27)	0.205	0.024	−2.22 (−12.60–8.15)	0.671	0.003	3.10 (5.45–−11.66)	0.472	0.008
YEC (mm)	−22.69 (−40.58–−4.80)	0.114	0.086	−1.71 (−16.76–13.32)	0.820	0.001	−1.04 (−15.4–13.40)	0.886	0.000	6.90 (−1.36–15.16)	0.100	0.039
LEC (mm)	−73.90 (−234.43–86.63)	0.362	0.012	33.75 (−102.82–170.32)	0.623	0.004	51.64 (5.34–97.94)	0.029	0.068	86.87 (7.98–165.75)	0.031	0.066
VEC (mm/s)	−7.07 (−13.68–−0.46)	0.360	0.063	−2.09 (−8.44–4.24)	0.511	0.006	−0.39 (−4.20–3.41)	0.837	0.001	3.91 (0.65–7.16)	0.019	0.178
SEC (mm^2^)	56.42 (−321.82–434.66)	0.767	0.001	110.40 (−313.15–533.95)	0.605	0.004	1.35 (−148.91–151.62)	0.986	0.000	83.59 (−34.34–201.54)	0.162	0.029

Values expressed as mean ± standard deviation and frequency (percentage). ABC: Activities Specific Balance Confidence Scale—16 items; DHI: Dizziness Handicap Inventory total score; EO: eyes open; EC: eyes closed; FD: frequency of dizziness; ID: intensity of dizziness; L: length of the stabilogram described by the movements of the center of pressure; S: surface described by the movements of the center of pressure; V: velocity of displacement of the center of pressure; X: deviation of the center of pressure: in the mediolateral directions; Y: deviation of the center of pressure in the anteroposterior axis.

## Data Availability

The data shown in this study are available upon request from the corresponding author. The data is not available to the public since, taking into account the sensitive nature of all the questions asked in this study, all participants were guaranteed that the data obtained would be confidential and would not be shared.

## Supplementary Materials

The following supporting information can be downloaded at: https://www.mdpi.com/article/10.3390/ijerph192215080/s1, Section S1. Graphical representation of the study design. Section S2. Questionnaires.
