# Supplementary material for: The Effects of Vestibular Rehabilitation and Manual Therapy on Patients with Unilateral Vestibular Dysfunction: A Randomized and Controlled Clinical Study
Source: Int J Environ Res Public Health. 2022 Nov 16;19(22):15080. doi: 10.3390/ijerph192215080 (PMC9690966; doi:10.3390/ijerph192215080)
Supplement: Supplementary file 1 [file ijerph-19-15080-s001.zip › ijerph-1987460-supplementary.pdf]

Supplementary Material Section S1. Graphical representation of the study design.

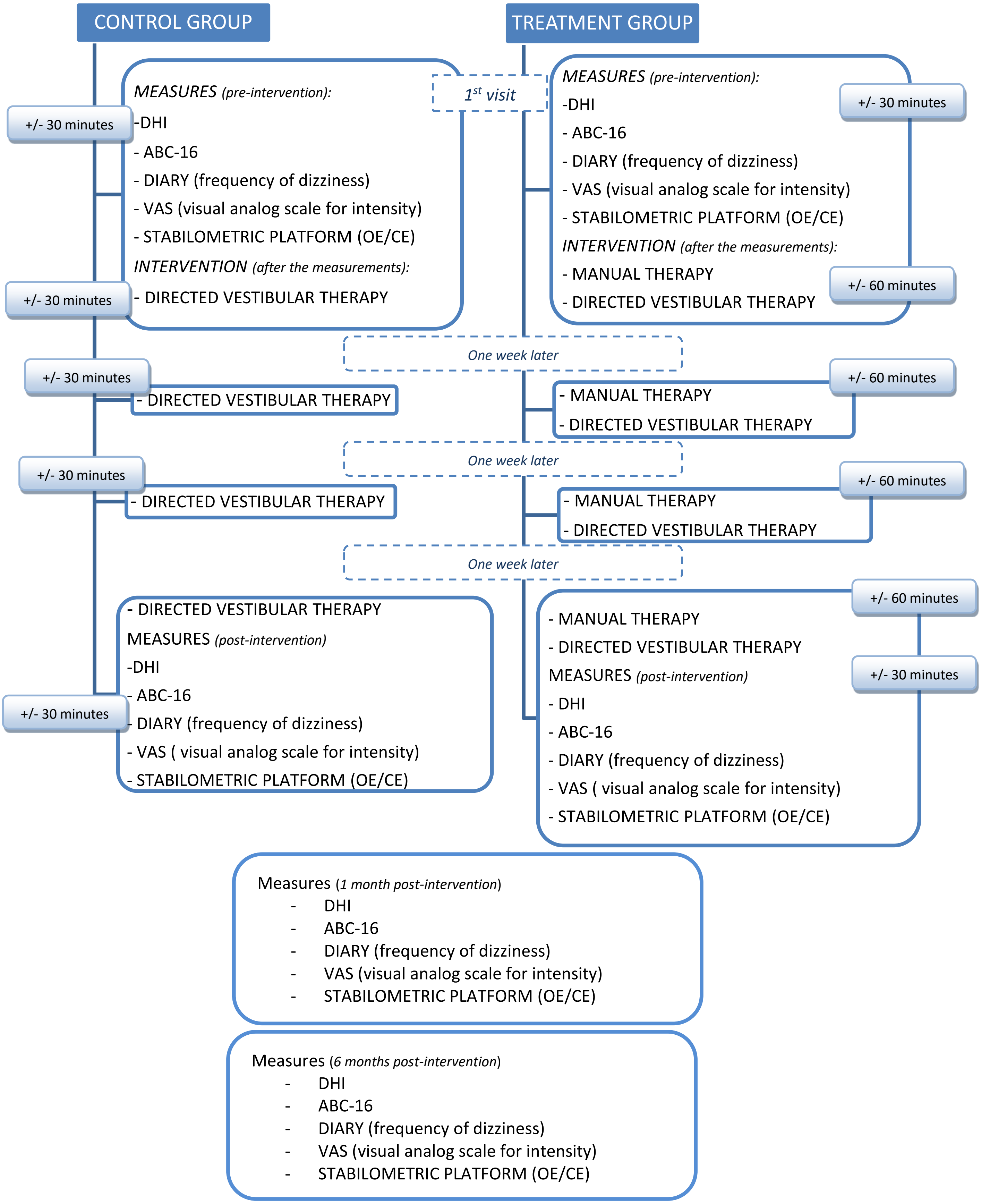

ABC-16, Activities Specific Balance Confidence Scale 16 items; DHI, Dizziness Handicap Inventory total score; OE, open eyes; CE: closed eyes; VAS, visual analog scale.

Supplementary Material Section S2. Questionnaires.

Escala ABC-16

Para cada una de las siguientes actividades, por favor indique su nivel de seguridad o confianza en sí mismo/a, escogiendo un número (porcentaje) de la siguiente escala de valoración:

0%	10	20	30	40	50	60	70	80	90	100%
Ninguna confianza										Confianza total

¿Cuánta confianza tiene en no perder el equilibrio o sentirse inestable cuando usted:"

1. Pasea por su casa? __________%
2. Sube o baja escaleras? __________%
3. Se agacha para alcanzar una zapatilla del suelo cerca de la puerta de un armario? __________%
4. Alcanza una lata pequeña de una estantería que se encuentra al nivel de los ojos? __________%
5. Se pone de puntillas y alcanza algo que se encuentra por encima de la cabeza? __________%
6. Se sube en una silla para alcanzar algo? __________%
7. Barre el suelo? __________%
8. Sale caminando de la casa hacia un coche aparcado en el garaje? __________%
9. Sube o baja del coche? __________%
10. Camina por el aparcamiento al centro comercial? __________%
11. Sube o baja una rampa o pendiente? __________%
12. Camina en un centro comercial lleno y la gente pasa a su alrededor con rapidez? __________%
13. Se topa o tropieza con la gente mientras anda por un centro comercial? __________%
14. Se sube o se baja de las escaleras mecánicas apoyándose en el pasamanos? __________%
15. Se sube o se baja de las escaleras mecánicas mientras lleva paquetes y no puede apoyarse en el pasamanos? __________%
16. Camina sobre aceras heladas? __________%

Cuestionario DHI

Instrucciones:

Por favor conteste "**si**", "**no**" o "**a veces**" a cada pregunta. Conteste a cada una de las preguntas según se refieran al problema de su vértigo o falta de equilibrio.

1. ¿Levantar la vista aumenta su problema? ________
2. ¿Se siente frustrado a causa de su problema? ________
3. A causa de su problema ¿decide limitar sus viajes de negocios o de ocio? _______
4. ¿Caminar por el pasillo de un supermercado aumenta su problema? ________
5. A causa de su problema ¿experimenta dificultades al acostarse y levantarse de la cama? ________
6. ¿Su problema limita de forma significativa su participación en actividades de ocio tales como cenar fuera de casa, ir al cine, ir a bailar o ir a fiestas? ________
7. A causa de su problema ¿experimenta dificultades al leer? ________
8. ¿Realizar actividades más exigentes tales como hacer deporte, bailar, o realizar trabajos domésticos (por ejemplo, barrer o recoger los platos) aumenta su problema? ________
9. A causa de su problema ¿tiene miedo a salir de casa sin que nadie le acompañe? _______
10. A causa de su problema ¿ha sentido vergüenza delante de otros? ________
11. ¿Los movimientos rápidos de cabeza aumentan su problema?
12. A causa de su problema ¿evita las alturas? ________
13. ¿Aumenta su problema al darse la vuelta en la cama?________
14. A causa de su problema ¿le resulta difícil realizar trabajos domésticos agotadores? ________
15. A causa de su problema ¿tiene miedo a que la gente piense que está ebrio? _______
16. A causa de su problema ¿le resulta difícil pasear solo? ________
17. ¿Caminar por la acera aumenta su problema? ________
18. A causa de su problema ¿le resulta difícil concentrarse? ________
19. A causa de su problema ¿le resulta difícil caminar por su casa a oscuras? _______
20. A causa de su problema ¿tiene miedo a quedarse solo en casa? ________
21. A causa de su problema ¿se siente deprimido? ________
22. ¿Su problema ha dificultado las relaciones con sus familiares o amigos? _______
23. ¿Influye negativamente su problema en sus responsabilidades domésticas o laborales? ________
24. ¿Su problema interfiere en su trabajo o en sus responsabilidades de familia? _______
25. ¿Aumenta su problema al agacharse? ________
